# Paralytic Shellfish Toxin Concentrations Measured in Alaskan Arctic Clams Using ELISA and HPLC Methods

**DOI:** 10.3390/toxins17020060

**Published:** 2025-01-28

**Authors:** Patrick Charapata, Emily K. Bowers, Donnie Ransom Hardison, Steve Kibler, Donald M. Anderson, Evangeline Fachon, Kathi A. Lefebvre

**Affiliations:** 1 Environmental and Fisheries Science Division, Northwest Fisheries Science Center, National Oceanic and Atmospheric Administration (NOAA), Seattle, WA 98112, USA; 2 Center for Species Survival, Georgia Aquarium, Atlanta, GA 30313, USA; 3 Beaufort Laboratory, National Ocean Service, National Oceanic and Atmospheric Administration, 101 Pivers Island Road, Beaufort, NC 28516, USA; 4 Biology Department, Woods Hole Oceanographic Institution, Woods Hole, MA 02543, USA

**Keywords:** paralytic shellfish toxins, Arctic, clams, Alaska, ELISA, HPLC, correction factors, saxitoxin, HAB

## Abstract

Clams are efficient vectors of potent algal neurotoxins, a suite of saxitoxin (STX) congeners collectively called paralytic shellfish toxins (PSTs), to higher trophic levels. The Alaskan Arctic is a region facing an expanding threat from PSTs due to ocean warming, yet little is known about PSTs in clams from this region. Quantifying total toxicity in bivalves requires analytical techniques, such as high-performance liquid chromatography (HPLC). Enzyme-linked immunosorbent assays (ELISAs) are an efficient but only semi-quantitative method for measuring clam toxicity. PSTs (STX eq.) were measured in split clam samples (*n* = 16) from the Alaskan Arctic using ELISA and HPLC methods to develop a preliminary linear model for conservatively estimating total toxicity in clams from ELISA toxin values (R^2^_adj_ = 0.95, *p* < 0.001). Profiles of PST congeners and total toxicity using HPLC were also assessed in additional clams (*n* = 36 additional, *n* = 52 total). Clams contained mostly potent PST congeners, and over half of the clams had PST concentrations above the seafood regulatory limit. These data will help assess the exposure risks of PSTs in Arctic marine food webs, as harmful algal bloom activity is predicted to increase as the Arctic continues to warm.

## 1. Introduction

Paralytic shellfish toxins (PSTs) are a suite of potent neurotoxins produced by the harmful algal bloom (HAB) species *Alexandrium catenella* that can cause negative impacts to human and wildlife health [1]. There are over 20 PST congeners of saxitoxin (STX) that share a similar alkaloid structure and are categorized into four different divisions based on functional group differences [2,3]. Paralytic shellfish toxins have a high affinity and easily bind to voltage-gated sodium ion channels in nerve and muscle cells, impairing central nervous system activity [3]. Thus, a congener’s potency or toxicity is analogous to how efficiently it binds to and inhibits sodium ion channel processes. Saxitoxin (STX), neosaxitoxin (NEO), and certain gonyautoxins (GTX1, 3, and 4) are the most potent congeners, while carbamate toxins (C-toxins) are the least potent [4]. Toxin production is variable across algal strains and under different environmental conditions [5], with broad-scale geographic differences [6] in the dominant toxin congeners produced by *A. catenella*. This wide variability, both in toxin production and in relative potency, reveals a need to differentiate among these congeners when assessing total toxicity (i.e., composite toxicity from all congeners present in a sample) in seafood for monitoring or research purposes. As a result, analytical methods to quantify PST congeners in shellfish have greatly evolved since testing began in the 1950s [7].

Historically, PST toxicity in seafoods for human consumption was quantified using either a mouse bioassay (MBA) [8], where seafood extracts were injected into mice and toxicity was related to death time, or through high-performance liquid chromatography with fluorescence detection (HPLC-FLD) [9]. While HPLC is a highly quantitative method of toxin detection, it is also time- and resource-intensive, requiring expensive lab equipment, a suite of certified reference standards, and an expert technician to properly analyze samples. When measuring toxicity through HPLC, the concentration of each PST congener is multiplied by a congener-specific toxicity equivalency factor (TEF), which is the relative potency of that toxin form compared to STX; the TEF-converted values of all congeners detected in the sample are combined to evaluate total toxicity [10]. Additional methods such as the receptor binding assay (RBA) [11] were developed either to replace (e.g., MBA), enhance, validate, supplement, or streamline these official methods [10,11,12,13,14,15]. More recently, liquid chromatography paired with mass spectrometry (LC-MS) methods have been validated to measure PSTs in shellfish, which either maintains or improves upon the high-quality analysis of HPLC while also streamlining certain aspects of HPLC (e.g., avoiding derivatizations) [16,17,18].

Commercially manufactured enzyme-linked immunosorbent assays (ELISAs) have also been used to semi-quantify PSTs in various marine food web samples [13,19,20,21,22,23]. ELISAs are an efficient method for analyzing PSTs in marine organisms because they are relatively cheap, quick, and straight-forward to perform in a lab. The largest drawback of ELISAs is that they regularly underestimate total toxicity due to low (or zero) cross-reactivity of the antibodies with some PST congeners [13,23]. Therefore, HPLC methods that provide full PST congener profiles are typically used to supplement, correct, and/or verify ELISA PST measurements [15,23,24].

Climate change in the Alaskan Arctic (Bering Strait [i.e., Northern Bering Sea], Chukchi Sea, and Beaufort Sea regions) has allowed the PST-producing dinoflagellate *Alexandrium catenella* to thrive during summer conditions, which has led to detectable PST concentrations in every trophic level of the marine ecosystem [22,23,25]. Specifically, benthic invertebrates (e.g., clams) have been identified as critical PST vectors to Arctic wildlife [23]. In a previous study using ELISAs, PST concentrations in Alaskan Arctic clams exceeded the seafood regulatory limit (80 µg STX eq. 100 g^−1^), but these were considered underestimates based on paired HPLC PST values [23]. Despite this drawback, the use of ELISA’s for screening large numbers of samples is an effective way to monitor food web contamination on a large scale. Further research is required, however, to characterize the degree of underestimation of total PST toxicity in benthic invertebrates from the Alaskan Arctic to fully assess their threat as vectors of PSTs to higher-trophic-level consumers, such as walruses and humans.

In the present study, clams were collected during 2019, 2020, and 2022 on Alaskan Arctic research cruises of opportunity for the analysis of PSTs (STX eq.) using ELISA and HPLC methods. It is known that ELISAs typically underestimate total toxicity in clams compared to HPLC methods due to variable reactivity between the antibody and the varying suite of PST congeners in different regions [13,23]. To better understand the PST threat in the region, it was also necessary to obtain detailed toxin profile information, since few data are available on the total toxicity of Alaskan Arctic clams [23,26,27]. The objectives of this study were as follows: (1) to characterize PST congener profiles (*n* = 51) and total toxicities (*n* = 52) in Arctic clams across different regions in the Alaskan Arctic using HPLC; (2) to compare PST concentrations (STX eq.) obtained using ELISA and HPLC methods in split clam samples (*n* = 16) collected from the region; and (3) to use the results of this comparison to develop a linear model that estimates total PST concentrations from ELISA quantifications in clams. Overall, this study is a first step for estimating total toxicity using rapid and simple ELISA measurements and contributes critical PST data in a key algal toxin vector from the Alaskan Arctic.

## 2. Results

### 2.1. Correction Model for ELISA STX Eq. Values

**Figure 1:**
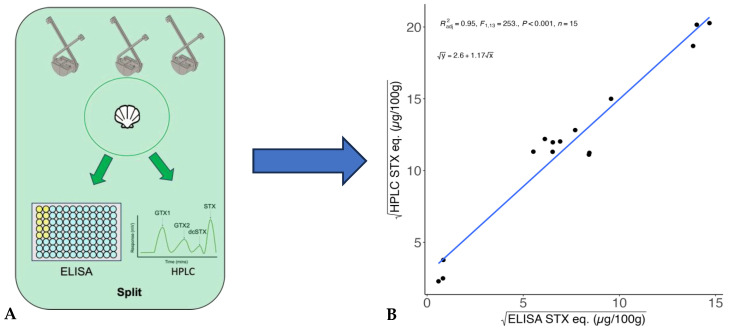
(**A**,**B**): (**A**) Schematic of sampling design for comparing STX eq. values in clams using ELISA and HPLC methods. Clams were collected from multiple stations (multiple Van Veen Grabs), pooled together, and the sample mass was split equally for ELISA and HPLC analysis. (**B**) Linear relationship between square root transformed HPLC and ELISA STX eq. concentrations calculated using toxicity equivalency factors (TEFs, µg STX eq. 100 g^−1^) (Table 2) in “split” clam samples (black points, *n* = 15). Linear trendline (blue) with linear regression results provided (linear model equation, adjusted R^2^ (R^2^_adj_), F statistics, *p* value, and sample sizes (*n*)). Van Veen grab illustration attributed to Hans Hillewaert and used unaltered under a Creative Commons use license (CC-BY-SA 4.0).

The STX eq. values obtained using ELISAs underestimated total toxicity in clams, detecting 5–57% of the toxicity measured by HPLC methods (Table 1). Linear regressions based on HPLC toxin profile results revealed that total PST concentrations can be estimated from ELISA STX eq. concentrations in Alaskan Arctic clams (Figure 1A,B; R^2^_adj_ = 0.95, *p* < 0.001, *n* = 15). The linear model is as follows: $\sqrt{Total\;PST\;toxicity\;(µg\;STX\;eq./100\;g)}=2.6+1.17\times \sqrt{ELISA\;STX\;eq.\left(µg\;STX\;eq./100\;g\right)}$ (Figure 1B).

**Table 1:** Paralytic shellfish toxin (PST) concentrations (μg STX eq./100 g) in clams (*n* = 15) determined using ELISA and HPLC methods. HPLC concentrations (μg STX eq. 100 g^−1^) are derived from the conversion of PST congeners to STX eq. values using toxicity equivalency factors (TEFs, Table 2). The resulting percent toxicity ELISA values compared to HPLC values (“ELISA% of HPLC”) and whether this comparison resulted in an underestimate or overestimate of toxicity are included (“ELISA +/− to HPLC”) for reference.

Species	Region	Date	ELISA (μg STX eq./100 g)	HPLC (μg STX eq./100 g)	ELISA% of HPLC	ELISA +/− to HPLC
*Carditidae sp.*	Beaufort Sea	7 August 2022	0.35	5.21	7	−
*Siliqua patula*	Beaufort Sea	7 August 2022	0.68	6.20	11	−
*Macoma calcarea*	Bering Strait	26 July 2022	0.71	14.22	5	−
*Macoma calcarea*	Chukchi Sea	11 August 2022	30.51	128.00	24	−
*Macoma calcarea*	Bering Strait	8 August 2019	37.47	148.80	25	−
*Macoma calcarea*	Chukchi Sea	11 August 2022	42.58	127.80	33	−
*Macoma calcarea*	Chukchi Sea	11 August 2022	42.71	143.20	30	−
*Macoma calcarea*	Bering Strait	18 October 2020	47.84	144.60	33	−
*Macoma calcarea*	Beaufort Sea	7 October 2020	59.28	164.30	36	−
*Macoma calcarea*	Chukchi Sea	24 November 2022	70.79	123.40	57	−
*Macoma calcarea*	Chukchi Sea	11 August 2022	71.18	126.20	56	−
*Macoma calcarea*	Bering Strait	18 October 2020	91.49	224.90	41	−
*Macoma calcarea*	Chukchi Sea	24 November 2022	191.29	348.90	55	−
*Macoma calcarea*	Chukchi Sea	24 November 2022	196.61	406.40	48	−
*Macoma calcarea*	Chukchi Sea	24 November 2022	215.57	410.90	52	−

**Table 2:** Summary of paralytic shellfish toxin congeners assessed in Alaskan Arctic clams. Cross-reactivities of Abraxis enzyme-linked immunosorbent assay (ELISA) with PST congeners are from the user manual (“-” indicates value not listed). Toxicity equivalency factors (TEFs) are those recommended by the European Food Safety Authority [4]. * STX represents the STX diHCL form of saxitoxin.

Congener	Molecular Weights [g/mol]	Cross-Reactivity (%)	TEF
STX *	372.2	100%	1.0
NEO	315.3	1.30%	1.0
GTX1	411.4	<0.2%	1.0
dcSTX	256.3	-	1.0
GTX4	411.4	<0.2%	0.7
GTX3	395.4	23%	0.6
dcGTX3	352.3	1.40%	0.4
GTX2	395.4	23%	0.4
dcGTX2	352.3	1.40%	0.2
GTX5	379.4	23%	0.1
C2	475.4	-	0.1
C1	-	-	0.1

### 2.2. PST Profiles and Total Toxicities of Alaskan Arctic Clams

The most potent PST congeners (STX and NEO) had the highest relative proportions in HPLC profiles from Arctic clams, but those profiles were highly variable. NEO had the highest proportions (23% ± 25%, mean ± SD, *n* = 51), followed by STX (22% ± 20%). Notable proportions of GTX2 (16% ± 18%) and GTX3 (16% ± 21%) were present in most clams, with minor contributions from other congeners (<10% per congener). These patterns mostly represent PST profiles of *Macoma calcarea* (*n* = 39 of 51). The remaining taxa of clams assessed were *Astarte borealis* (*n* = 2), *Carditidae* sp. (*n* = 1), *Ennucula tenuis* (*n* = 3), *Siliqua patula* (*n* = 2), *Serripes notabilis* (*n* = 1), and *Veneridae* sp. (*n* = 3).

**Figure 2:**
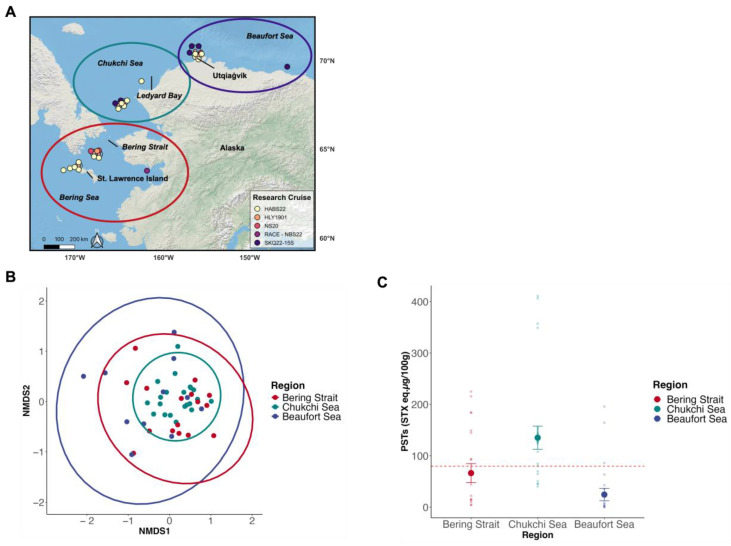
(**A**–**C**): (**A**) Map of stations where clams were collected during research cruises of opportunity (colored points) with the larger colored circles representing different Alaskan Arctic regions (red: Bering Strait; green: Chukchi Sea; and purple: Beaufort Sea). (**B**) Non-metric dimensional scaling (NMDS) plots visualizing regional similarities of paralytic shellfish toxin (PST) profiles (i.e., relative molarity; [%] of each congener) in Arctic clams (*n* = 51). Points represent each clam and are colored by region (Bering Strait [*n* = 16], Chukchi Sea [*n* = 22], and Beaufort Sea [*n* = 13]). Colored ellipses represent the 95% confidence interval for each region. (**C**) PST profiles were converted to STX eq. concentrations using toxicity equivalency factors (TEFs, Table 2) (*n* = 52) to compare estimated marginal mean ± 1 SE total PSTs (µg STX eq. 100 g^−1^) (large colored points) and individual clam PSTs (smaller more transparent points) within and among each region (note: Beaufort Sea, *n* = 14). Seafood regulatory limit (80 µg STX eq. 100 g^−1^) plotted for reference (red dashed line).

**Figure 3:**
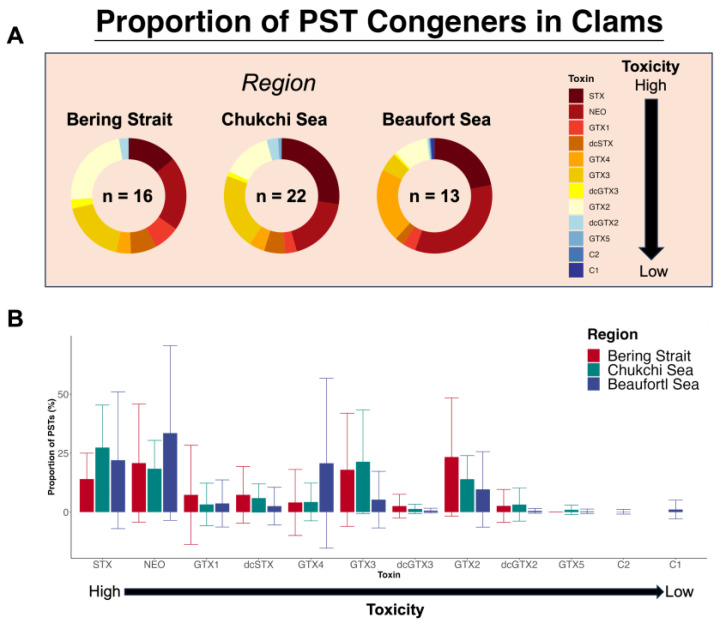
(**A**–**B**): (**A**) Overall regional comparison of mean proportions (i.e., relative molarity; [%] of each congener) of paralytic shellfish congeners (red–blue scale bar) in Arctic clams. Sample sizes (*n*) for each region are located inside donut plots. (**B**) Variation in each congener present in each region. Specifically, the mean ±1 SD congener abundances (relative molarity; [%] of each congener) measured in Arctic clams are plotted by region (red: Bering Strait; green: Chukchi Sea; purple: Beaufort Sea). Congeners are ranked from high to low toxicity (black arrows) based on their toxicity equivalency factors (TEFs, Table 2).

Clams collected from different regions (Bering Strait, Chukchi Sea, and Beaufort Sea) exhibited variable PST profiles. There were no strong regional groupings based on overall PST profiles (ANOSIM, R = 0.19, Figure 2A,B); however, there were significant regional dissimilarities among abundances of specific PST congeners (ANOSIM, *p* < 0.001). Clams from the Bering Strait and Chukchi Sea had similar toxin profiles (SIMPER, Table 3). The Bering Strait clams were dominated by GTX2 (23% ± 25%), NEO (21% ± 25%), GTX3 (18% ± 24%) and STX (14% ± 11%) (Table 3 and Figure 3A,B). Clams from the Chukchi Sea primarily contained STX (27% ± 18%), GTX3 (21% ± 22%), NEO (18% ± 12%) and GTX2 (14% ± 10%) (Table 3 and Figure 3A,B). Clams from the Beaufort Sea had dissimilar abundances of congeners compared to clams from the Bering Strait and Chukchi Sea (Table 3). Specifically, Beaufort Sea clams contained higher proportions of NEO (34% ± 37%) and GTX4 (21% ± 36%) compared to Bering Strait and Chukchi Sea clams, while having lower proportions of GTX2 (10% ± 16%) compared to Bering Strait clams and lower proportions of STX (22% ± 29%) compared to Chukchi Sea clams (Table 3 and Figure 3A,B).

**Figure 4:**
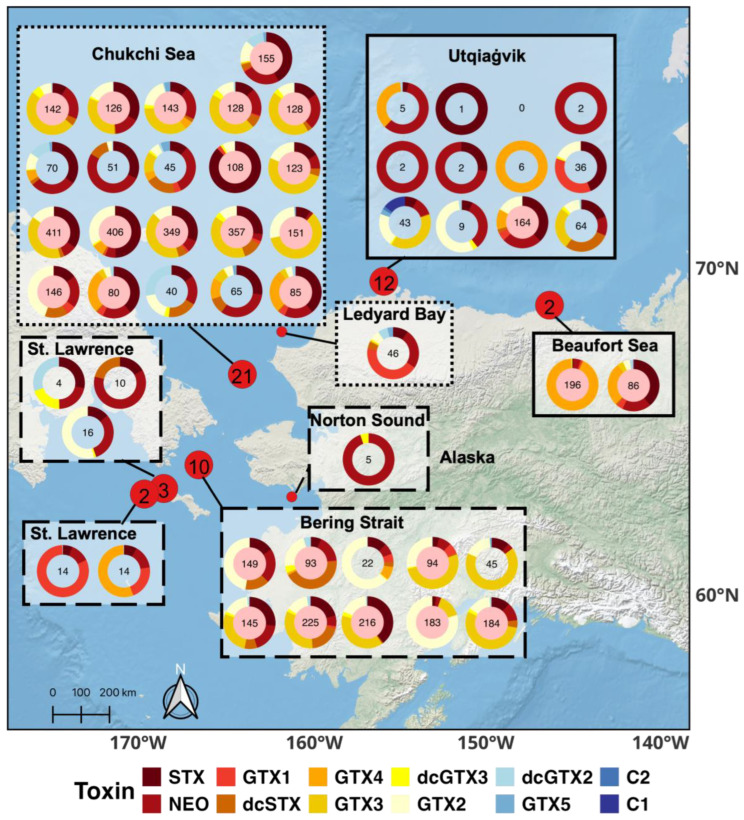
Map of all clams’ (*n* = 52) paralytic shellfish toxin (PST) congener profiles (i.e., relative molarity [%] of each congener) from HPLC analysis with the total toxicity concentration (µg STX eq. 100 g^−1^) of each clam contained inside the donut plot. Each congener in each PST profile was converted to STX eq. concentrations using toxicity equivalency factors (TEFs, Table 2) and then added together for each clam, resulting in their total toxicity concentration (µg STX eq. 100 g^−1^). Red points on the map depict locations where clams were collected and number inside each point correlates to the number of clams collected in that area. Borders of boxes reflect the region each clam was collected from, including the Bering Strait (long-dashed borders, *n* = 16), Chukchi Sea (short-dashed borders, *n* = 22), and Beaufort Sea (solid-line borders, *n* = 14). Donut plots with red centers are clams with total PST concentrations above the seafood regulatory limit (≥80 µg STX eq. 100 g^−1^).

Toxicity (µg STX eq. 100 g^−1^, HPLC analysis) above the seafood regulatory limit of 80 µg STX eq. 100 g^−1^ was documented in clams throughout the Alaskan Arctic (Figure 4). Paralytic shellfish toxin concentrations (EMM ± SE) were significantly different among regions based on the model selection process and subsequent statistical analysis (ANOVAs, *p <* 0.001). Clams collected in the Chukchi Sea (135 ± 23 µg STX eq. 100 g^−1^) had significantly higher PSTs than clams in the Beaufort (25 ± 12 µg STX eq. 100 g^−1^), but not the Bering Strait (58 ± 17 µg STX eq. 100 g^−1^) (Tukey HSD, *p <* 0.001; Beaufort Sea, *p =* 0.06; Bering Strait); clams from the Bering Strait and Beaufort Sea had similar PST concentrations (Tukey HSD, *p =* 0.14) (Figure 2C). Overall, over half of the clams collected (52%, *n =* 27 of 52) throughout the Alaskan Arctic were above the seafood regulatory limit, with a maximum toxicity of 411 µg STX eq. 100 g^−1^ (Figure 4).

## 3. Discussion

This study aims to characterize PST profiles and toxicities in relatively unstudied Alaskan Arctic clams and to use that data to develop a method to estimate total PST concentrations (STX eq.) from ELISA STX eq. concentrations. ELISA analyses are faster and simpler, but tend to underestimate total toxicity because of variable reactivity between antibodies and the suite of PST toxins. That was indeed observed in this study where ELISA values were consistently lower than paired values obtained using HPLC and had high variability in detecting total PSTs in clams (between 5 and 57% of total toxicity measured, Table 1). These results were used to develop a highly significant linear model to conservatively estimate total PST concentrations from ELISA STX eq. data. Given the limited sample size for the linear model (*n* = 15), application of this model to an independent (i.e., “testing”) set of clams would improve model validation. The PST profiles revealed that toxic clams throughout the Alaskan Arctic generally contained highly potent PST congeners, and that toxin profiles in clams from different regions were variable. The corrective linear model can be used to provide better estimates of total clam toxicity from ELISA methods, and additional clam PST data will contribute to the understanding of PST dynamics in Arctic food webs that are experiencing a rapidly warming ecosystem [28].

### 3.1. Linear Model to Estimate Total PST Toxicity from ELISA STX Eq. Measurements

A linear model was developed to estimate total PST toxicity from ELISA STX eq. measurements in Arctic clams. Clams from low to high toxicity (0.35–216 µg STX eq. 100 g^−1^ [ELISA]; 5–411 µg STX eq. 100 g^−1^ [HPLC]) were used to develop the linear regression model to estimate total PSTs from ELISA values (R^2^_adj_ = 0.95, Figure 1B). Similar comparisons have shown that ELISA and HPLC STX eq. concentrations are strongly correlated in paired clam samples [19,24,29], but that ELISAs underestimate total toxicity (Table 1). ELISAs in this study underestimated total toxicity by 43–95% (Table 1) in Alaskan Arctic clams. The Abraxis kit does not have significant cross-reactivity with certain potent congeners such as NEO (1.3%) or GTX1 and 4 (<0.2%) (Table 2). This would explain the underestimated total toxicity in these samples (Table 1) considering that NEO is one of the most abundant and most toxic congeners in the clam samples from this study (Figure 3A,B). Alaskan Arctic clams also had prominent levels of GTX2 and GTX3, which have cross-reactivity levels of ~23%, leading to additional total toxicity underestimation. This simple but effective model allows researchers to acquire a conservative estimate of total toxicity in Alaskan Arctic clams using the extraction methods from this study and a commercially manufactured (Abraxis) ELISA kit. This model only applies when toxin extraction and measurement methods from this study are followed because different extraction methods and/or ELISA kits are likely to produce different results [29,30]. Application of the linear model to an independent (i.e., “testing”) sample set of clams would improve model validations given the limited sample size (*n* = 15) and high variability in the ELISA’s ability to measure total toxicity in Alaskan Arctic clams (Table 1).

There was one clam with a below detectable limit (BDL) value using the ELISA, whereas its HPLC concentration was 86 µg STX eq. 100 g^−1^. The contribution of STX to this clam’s PSTs was ~39%, which should have been detectable on this ELISA, resulting in its exclusion from the linear regression analysis. False negatives have been documented in other clams using the Abraxis kit, which were mostly due to clams containing an abundance of congeners that were not detectable by the kit, such as GTX 1 and 4 [29,30]. This outlier sample did have a significant abundance of GTX 1 and 4 (~31%), which could have resulted in the false negative. This result demonstrates that high-throughput studies assessing STX eq. in clams using ELISAs would benefit from allocating a subset of clams for QA/QC purposes using HPLC methods. Additionally, the outlier clam shows that the model will underestimate total toxicity if it is applied to clams that exhibit different PST congener profiles to those that were included in the linear model analysis (Figure 1A,B).

### 3.2. Paralytic Shellfish Toxin Profiles in Alaskan Arctic Clams

Clams from the Alaskan Arctic contained primarily highly toxic PST congeners. STX (22% ± 20%, mean ± SD) and NEO (23% ± 25%) together comprised almost half of the congener proportions in all clams (Figure 3A,B and Figure 4). Most clams were collected during an extensive and highly toxic *Alexandrium* bloom (>1000 cells L^−1^) which extended through the Bering Strait and into the eastern Chukchi Sea in the summer of 2022 (Table 1, Figure 2A [HABS2022 cruise]) [31]. *Alexandrium* cell densities in surface waters during the 2022 bloom were documented at >5000 cells L^−1^ in certain parts of the Bering Strait region during late July, while reaching peak densities >174,000 cells L^−1^ in the southern Chukchi Sea during late August [31]. The *Alexandrium* from this bloom contained comparable proportions of NEO (21 ± 10%), but not STX (9 ± 4%), compared to clams in this study (Figure 3A,B); Gonyautoxin-4 (GTX4) was the most abundant congener (49% ± 11%) in the *Alexandrium* congener suite [31]. Possibly, bioconversion of congeners (e.g., STX and GTX4) by clams after consuming cells [32], differential uptake or retention of toxin congeners [33], or methodological differences when extracting and measuring the suite of PSTs in the *Alexandrium* [15] compared to those used in this study could explain the differences in congener abundances between *Alexandrium* and clams during this bloom event. Bioconversion of congeners was suggested as a likely mechanism for the different PST profiles among paired *Alexandrium* and *M. calcarea* samples collected from similar Arctic regions during the summer of 2019 [23]; however, the metabolism of congeners by Alaskan Arctic clams has not been directly tested and warrants further research.

Proportions of PSTs were dissimilar in clams across different Arctic regions in Alaska. Beaufort Sea clams had different congener abundances compared to Chukchi Sea and Bering Strait clams. These are similar results found with a limited samples of *M. calcarea* analyzed for PSTs in 2019, where qualitative differences in PST profiles were observed across regions [23]. Similar trends were found relating to Bering Strait clams containing less STX compared to Chukchi Sea clams [23] (Figure 3A,B). As a geographic group, Chukchi Sea clams had the least variability (i.e., most similarity) in PST profiles compared to Bering Strait and Beaufort clams (Figure 2B). This could indicate these clams are exposed to a consistent source of *Alexandrium*, whereas Bering Strait and Beaufort Sea clams may be exposed to different or multiple strains of *Alexandrium* that produce different PST profiles [31,34,35]. *Alexandrium* bloom patterns in the Alaskan Arctic are complex given the warming environment [25,31,34,36], making it difficult to isolate drivers in clam toxin profiles across regions with the sample sizes in this study.

### 3.3. Total Toxicity of Alaskan Arctic Clams

The PST congener profiles equated to high PST concentrations (>80 µg STX eq. 100 g^−1^) found in clams from all Alaskan Arctic regions (Figure 4). Significant bloom activity (>5000 cells L^−1^) has been documented in the Bering Strait region [31], which is then advected into the Chukchi Sea leading to significant deposition of *A. catenella* resting cysts in the Ledyard Bay Region [25,37]. These dormant resting cysts accumulate in sediments of the Chukchi Shelf and can serve as an inoculum for blooms under conducive environmental conditions. While the degree to which bivalves may ingest these *Alexandrium* cysts and retain their toxins is not well understood, this phenomenon has been observed [38,39]. Therefore, PST accumulation of resting cysts on the Chukchi Shelf could provide a readily available year-round source of algal toxins to Arctic clams, potentially explaining how Chukchi Sea clams had the highest PST concentrations (Figure 2C). While there has been relatively less bloom activity documented in the Beaufort Sea [25,31], clams were sampled near Barrow Canyon, the site of a relatively small (128,100 km^2^; Ledyard Bay, 17,500 km^2^; Barrow Canyon) but dense cyst bed (~2068 cm^−3^) [25]. Clams collected near Barrow Canyon could obtain high toxicities through cyst consumption [38,39] or by consuming newly germinated cells originating from these cysts during the bloom season [25,37,40]. While these are plausible explanations for clam toxicity patterns in the Alaskan Arctic, toxicity varied greatly across and within regions (Figure 2C and Figure 4). Consequently, concrete conclusions about PST toxicity patterns in Arctic clams cannot be made given the limited sample sizes in different regions. Furthermore, other factors contribute to variability in clam toxicity that were not addressed in this study, including differences in collection dates (i.e., seasonality) [41], *Alexandrium* consumption rates, and PST depuration rates [42,43]. These results add to the growing data on total clam PST concentrations in the Arctic [23,26,27].

## 4. Conclusions

A corrective linear regression model was developed to conservatively estimate total PST toxicity (STX eq.) from ELISA STX eq. concentrations and PST congener profiles and toxicities were characterized in Alaskan Arctic clams. It is well known that liquid chromatography is a robust method to acquire total PSTs in samples, however, it is expensive, time consuming, and requires specialized materials and expertise. This model (Figure 1B) will allow researchers to better estimate total PST toxicity in Alaskan Arctic clams using ELISA methods, allowing quantification of large numbers of samples for ecosystem and exposure risk studies. Given that the corrective model presented in this study was derived from clams collected across multiple seasons and regions in three different years of study with variable bloom conditions, there can be confidence in applying the conversion to future data collection in the Alaskan Arctic based on the strong and highly significant relationships among ELISA and HPLC PSTs measured in this study (Figure 1A,B). It should be noted that the model predicts a conservative estimate of total PST toxicity for Alaskan Arctic clams due to the high variability of total toxicity the ELISA detected in this study (5–57%) and the use of a relatively small sample set (*n* = 15 clams). Thus, additional application to an independent set of clams would further validate the corrective linear model. ELISAs clearly underestimate total toxicity in Alaskan Arctic clams, thereby continued HPLC measurements of a subset of clams is still recommended for QA/QC purposes during high throughput studies of PST concentrations in clams using ELISAs. The PST profiles were characterized in Alaskan Arctic clams, for which there are limited data [23,26,27]. Paralytic shellfish toxin profiles indicated high variability among congener proportions in Arctic clams across Alaska that could only partially be explained by regional differences. PST profiles contained potent congeners that resulted in toxic clams (>80 µg STX eq. 100 g^−1^) documented in all sampled regions.

*Alexandrium* blooms in the Alaskan Arctic are predicted to continue and increase in frequency, intensity, and duration due to a warming climate [37]. This threatens the health of the entire ecosystem, including marine mammal populations that are utilized for subsistence purposes by Native Alaskan communities. The lasting effects these massive bloom events have on the health of the marine food web are unclear and require additional research as the Arctic continues to warm.

## 5. Materials and Methods

### 5.1. Sample Collection

Clams were collected during research cruises of opportunity (*n =* 5) during 2019, 2020, and 2022 along routinely sampled survey transects (Figure 2A). Van Veen Grabs were deployed at various stations along survey transects to collect clams from the sea floor. Grab contents were sieved with seawater to isolate clams, which were then sorted to the genus or species level, collected in sterile whirl-paks or zip-lock plastic bags, and stored at −20 °C until toxin analysis.

Clams were analyzed for PSTs using ELISA and HPLC methods based on sample availability at research cruise stations throughout the Alaskan Arctic (Figure 2A). Initially, one clam at each station was analyzed for PSTs (STX eq.) using an Abraxis^®^ ELISA kit (Gold Standard Diagnostics, Horsham, PA, USA). Based on ELISA results, stations throughout the Alaskan Arctic with clams containing low (BDL) to high toxins (≥80 µg STX eq. 100 g^−1^) were selected, and an additional clam sample (*n =* 1) was submitted for HPLC analysis. Additionally, one or more clams were pooled from either the same station or among similar stations along the same transect, homogenized using metal scissors and a spatula, and then the homogenate (*n =* 1) was split equally to perform ELISA (target sample mass; 1.0 g, minimum; 0.4 g) and HPLC (target mass; 1.0 g, minimum; 0.5 g) analyses (“split” sample, Figure 1A). These “split” samples were limited (*n* = 16) due to sample availability and achieving soft tissue mass required to perform both ELISA and HPLC analyses. A total of 52 samples were submitted for toxin analysis (*n =* 36, HPLC-only; *n =* 16, split samples for ELISA and HPLC analyses).

### 5.2. Paralytic Shellfish Toxin Measurements

#### 5.2.1. Abraxis ELISA

Saxitoxin (STX eq.) was measured in clam samples using an Abraxis^®^ ELISA kit (product no: 52255B) following Lefebvre et al. [22,23]. Solvent (50% methanol/50% water) was added to homogenized samples using a 4:1 ratio (solvent (mL)/sample mass (g)), which were then homogenized for 1 min at 2100 rpm using a hand-held probe (GLH 850, 10 mm; Omni-International, Kennesaw, GA, USA). Samples were then centrifuged at 3063× *g* for 20 min at 4 °C (Jouan CR3i centrifuge, Thermo Electron Corp., Waltham, MA, USA), extract transferred to 4 mL amber vials and stored at 4 °C prior to toxin analysis. Aliquots (200 µL) of extracts were filtered through a spin filter (Millipore Sigma Ultra-Free Centrifugal filters, 0.22 μm; Merck KGaA, Darmstadt, Germany) and further diluted to 1:50 prior to measuring STX concentrations (STX eq.) using the Abraxis^®^ ELISA kit. All manufacturer instructions were followed to obtain STX eq. concentrations (µg STX eq. 100 g^−1^) from extracts. The cross-reactivity of the ELISA antibodies with PST congeners according to the Abraxis user manual are listed in Table 2. ELISA values that were below detectable limits (BDL) (*n* = 2 clams) were included in linear model development by assigning half the lowest quantifiable limit (LQL) (LQL = 0.70 µg STX eq. 100 g^−1^, BDL= 0.35 µg STX eq. 100 g^−1^) [44,45].

#### 5.2.2. HPLC Methods

HPLC analysis of samples for paralytic shellfish toxins followed the post-column oxidation (PCOX) AOAC Official Method 2011.02 [46]. Extractions involved using equal volume of extraction acid to sample weight (1:1 ratio). Briefly, extraction acid (0.1N HCl) was added to a homogenized sample in a 50 mL Falcon centrifuge tube (Falcon – BD, Franklin Lakes, NJ) and placed in boiling water for 5 min. Afterwards, the cooled sample was spun down via centrifugation, and proteins were removed by adding 30% trichloroacetic acid (TCA) followed by pH adjustment with NaOH. After filtration of the processed sample, it was ready for analysis.

The HPLC instrumentation was a Waters Acquity Arc system equipped with a refrigerated autosampler (4 °C) and a Waters 2475 FLR fluorescence detector (Waters, Milford, MA, USA). All extracts were injected at 10 µL and a flow rate of 0.8 mL/min. Attached to the HPLC was a Pickering Laboratories ONYX PCX post-column oxidation instrument (Pickering Laboratories Inc., Mountain View, CA, USA). Flow rates for both oxidant and acid were 0.4 mL/min, and column temperature was 30 °C. Standards used were Certified Reference Material (CRM) obtained from the National Research Council Canada (NRCC, Ottawa, ON, Canada). Toxin analogs analyzed were the following: GTX1, GTX4, GTX2, GTX3, dcGTX2, dcGTX3, GTX5, NEO, dcSTX, STX, C1, and C2. The saxitoxin standard used was saxitoxin dihydrochloride, and all results are expressed as STX·diHCl equivalents. Detection limit was established as 2 µg STX·diHCl 100 g^−1^. Quality control of instrument and method performance was evaluated in several ways. Upon instrument startup, upper-level and lower-level calibration mix standards were run to ensure instrument response was sufficient. Then, after analysis of 4–6 samples, a mid-level standard mix was run to check that instrument performance was being maintained and not degraded. Post analysis, again, included analysis of upper-level and lower-level quality control standard mixes. Also, once a zero toxin sample was obtained, it was spiked with a standard mix and carried out through extraction and analysis to ensure no matrix effects were occurring by comparing those results with previous quality control standard mixes. Chromatograms for standard mixes and detectable congeners in a clam sample can be found in the Supplementary Material. Toxicity equivalency factors (TEFs) used in the equation below are listed in Table 2.

Calculations were carried out using the following equation to yield µg STX·diHCl eq. 100 g^−1^:

$$\mu g\;STX\cdot diHCl\;eq=\mu M\;toxin\times TEF\times \frac{372.2\;g}{1000\;mL}\times \frac{Fvol}{Ext.vol}\times \left(\frac{Wt+Vol}{Wt}\right)\times 100$$

where:

µM = concentration of toxin in extract;

Fvol = final volume of deproteinated extract (560 µL);

Ext. vol = volume of crude extract used (500 µL);

Wt = weight of sample used (g);

Vol = volume of acid extractant used (mL);

TEF = toxicity equivalence factor (Table 2).

The µg STX·diHCl eq. 100 g^−1^ concentrations from HPLC analysis are referred to as µg STX eq. 100 g^−1^ throughout the manuscript (including figures and tables) for ease in comparison of total toxicity concentrations across studies (e.g., EFSA [4]).

### 5.3. Statistical Analysis

All statistical analyses were conducted using the software programs R version 4.4.2 [47] and R Studio version 2024.09.01+394 [48]. All maps were generated using the software QGIS version 3.34 [49]. All samples (*n* = 52) analyzed for PSTs via HPLC were included in total toxicity analysis; however, one clam had zero measured toxins and was excluded from PST congener profile analyses (*n =* 51). An alpha of 0.05 was used as a significance threshold for all relevant analyses.

#### 5.3.1. Correction Model—ELISA and HPLC PST Measurements

An initial linear regression was performed using split clam samples (*n* = 16) to predict total PST concentrations (µg STX eq. 100 g^−1^, HPLC PST values) from ELISA STX eq. values. One clam was BDL for STX eq. using ELISA, but had a paired HPLC value of over 80 µg STX eq. 100 g^−1^. This clam was a clear outlier and was removed from linear regression analysis, resulting in a total of *n* = 15 clams for model development (Table 1). Linear models were compared with square root transformed and non-transformed data using AIC criteria to determine best model fit [50]. The selected model was verified for underlying assumptions by plotting model residuals with fitted values, covariates, and assessing the distribution of residuals [51].

#### 5.3.2. Paralytic Shellfish Toxin Profiles and Total Toxicity Analyses

Paralytic shellfish toxin profiles for clams were constructed by using relative abundances (µM, molarity) of each congener measured during HPLC analysis. Profiles were assessed for similarities among regions using distance-based multi-variate analysis. Specifically, toxin profiles of clams (*n =* 51) were compared among different regions (Bering Strait [*n =* 16], Chukchi Sea [*n =* 22], and Beaufort Sea [*n =* 13]) using non-metric multidimensional scaling (NMDS), analysis of similarities (ANOSIM), and similarity percentage (SIMPER) analyses from the R package *vegan* [52]. A Bray–Curtis transformation was applied to congener proportions in clams to calculate a distance matrix for NMDS, ANOSIM, and SIMPER analysis, which allowed for the assessment of which proportional differences of specific congeners contributed to overall dissimilarities in PST profiles among regions.

Differences in total toxicity (µg STX eq. 100 g^−1^, HPLC PST values) in clams collected from different regions was tested by comparing linear models with and without (i.e., null model) region as a main effect and selecting the model that had the lowest AICc value and highest AIC weight [50]. Models were generated using the R package *lme4* [53]. A square root transformation was applied to toxicity data to achieve normality (Shapiro–Wilks test, *p =* 0.08) for linear model analysis. The full and selected models were verified as described above for linear regression analysis. If the main effect (region) was selected, a Type II ANOVA (F-tests for linear models) with subsequent Tukey HSD tests compared estimated marginal mean (EMM) toxicity concentrations using the R package *emmeans* [54]. Estimated marginal means were compared because they are dependent on model results (compared to ordinary means based on empirical data) and, therefore, take into account unbalanced sample sizes across regions [54].

## Figures and Tables

**Table 3:** Similarity percentage (SIMPER) analysis results comparing dissimilarities in paralytic shellfish toxin (PST) congener profiles (i.e., relative molarity; [%] of congeners) in clams (*n* = 51) by region (Bering Strait [*n* = 16], Beaufort Sea [Beaufort, *n* = 13], and Chukchi Sea [Chukchi, *n* = 22]). Comparison (A:B) column states which region’s profiles were compared and each toxin’s mean percent contribution ±1 standard deviation to total dissimilarity in congener profiles between regions. The mean percent abundance for each region (A or B) are presented for reference. Significant contributions to dissimilarities in toxin profiles among region comparisons are depicted with a bolded *p* value. * Indicates small sample sizes in group comparisons (*n* = 1 clam with detectable C1/C2 congeners), and statistical comparisons are excluded but results are included for reference.

Comparison (A:B)	Toxin	Mean Percent (%) Contribution	Standard Deviation (%)	Mean Percent Abundance (%) Group A	Mean Percent Abundance (%) Group B	*p*
Bering Strait:Beaufort	NEO	16	15	21	34	**0.01**
Bering Strait:Beaufort	GTX2	11	11	23	10	**0.03**
Bering Strait:Beaufort	GTX4	11	17	4	21	**0.02**
Bering Strait:Beaufort	STX	10	11	14	22	0.43
Bering Strait:Beaufort	GTX3	10	11	18	5	0.90
Bering Strait:Beaufort	GTX1	5	10	7	4	0.37
Bering Strait:Beaufort	dcSTX	4	6	7	2	0.38
Bering Strait:Beaufort	dcGTX2	1	4	3	0	0.79
Bering Strait:Beaufort	dcGTX3	1	2	2	1	0.45
Bering Strait:Beaufort	C1	1	2	0	1	NA *
Bering Strait:Beaufort	GTX5	0	0	0	0	0.99
Bering Strait:Beaufort	C2	0	0	0	0	NA *
Chukchi:Beaufort	NEO	15	14	18	34	**0.04**
Chukchi:Beaufort	STX	13	11	27	22	**0.03**
Chukchi:Beaufort	GTX4	11	16	4	21	**0.02**
Chukchi:Beaufort	GTX3	11	10	21	5	0.64
Chukchi:Beaufort	GTX2	7	6	14	10	0.92
Chukchi:Beaufort	dcSTX	4	4	6	2	0.77
Chukchi:Beaufort	GTX1	3	6	3	4	0.81
Chukchi:Beaufort	GTX5	1	1	1	0	0.19
Chukchi:Beaufort	C1	1	2	0	1	NA *
Chukchi:Beaufort	C2	0	0	0	0	NA *
Chukchi:Bering Strait	GTX3	12	10	21	18	0.08
Chukchi:Bering Strait	GTX2	10	9	14	23	0.09
Chukchi:Bering Strait	STX	9	8	27	14	0.86
Chukchi:Bering Strait	NEO	9	10	18	21	0.98
Chukchi:Bering Strait	GTX1	5	10	3	7	0.38
Chukchi:Bering Strait	dcSTX	4	5	6	7	0.18
Chukchi:Bering Strait	GTX4	4	7	4	4	0.98
Chukchi:Bering Strait	dcGTX2	2	4	3	3	0.17
Chukchi:Bering Strait	dcGTX3	1	2	1	2	0.06
Chukchi:Bering Strait	GTX5	0	1	1	0	0.33
Chukchi:Bering Strait	C1	0	0	0	0	NA *
Chukchi:Bering Strait	C2	0	0	0	0	NA *

## Data Availability

The original data presented in the study are openly available in Dryad at https://doi.org/10.5061/dryad.ngf1vhj4s.

## Supplementary Materials

The following supporting information can be downloaded at: https://www.mdpi.com/article/10.3390/toxins17020060/s1, Figure S1: A HPLC chromatogram showing the peaks of the paralytic shellfish toxin standards, excluding C toxins (see Figure S3), used in this study. Standards are Certified Reference Materials (CRM) obtained from National Research Council Canada (NRCC). Toxin analogs analyzed were the following: GTX1, GTX4, GTX2, GTX3, dcGTX2, dcGTX3, GTX5, NEO, dcSTX, and STX; Figure S2: A HPLC chromatogram from a clam sample that contained quantifiable amounts of GTXs and saxitoxin (STX).; Figure S3: A HPLC chromatogram showing the peaks of the C toxin (C1 and C2) standards used in this study. Standards used were Certified Reference Material (CRM) obtained from National Research Council Canada (NRCC).

## Abbreviations

The following abbreviations are used in this manuscript:

PSTs	Paralytic shellfish toxins
ELISA	Enzyme-linked immunosorbent assay
HPLC	High-performance liquid chromatography
TEF	Toxicity equivalency factors
STX	Saxitoxin
NEO	Neosaxitoxin
GTX1–4	Gonyautoxins 1–4
dcGTX2–3	Decarbamoyl gonyautoxin 2–3
dcSTX	Decarbamoyl saxitoxin
C1–2	Carbamate toxins
Eq.	Equivalents
HAB	Harmful algae bloom
MBA	Mouse bioassay
BDL	Below detectable limit
RBA	Receptor binding assay
