# Supplementary material for: Paralytic Shellfish Toxin Concentrations Measured in Alaskan Arctic Clams Using ELISA and HPLC Methods
Source: Toxins (Basel). 2025 Jan 28;17(2):60. doi: 10.3390/toxins17020060 (PMC11860261; doi:10.3390/toxins17020060)
Supplement: Supplementary file 1 [file toxins-17-00060-s001.zip › toxins-3408458-supplementary.pdf]

Standard mix

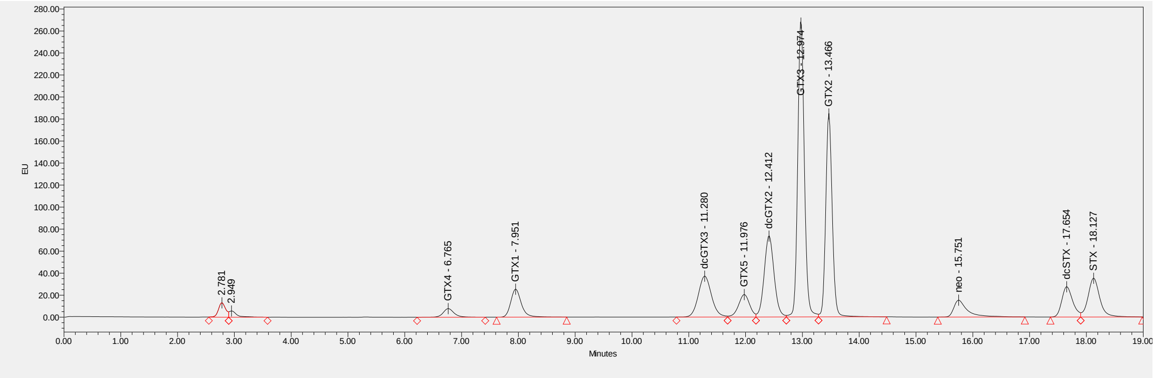

Figure S1: A HPLC chromatogram showing the peaks of the paralytic shellfish toxin standards, excluding C toxins (see Figure S3), used in this study. Standards are Certified Reference Materials (CRM) obtained from National Research Council Canada (NRCC). Toxin analogs analyzed were the following: GTX1, GTX4, GTX2, GTX3, dcGTX2, dcGTX3, GTX5, NEO, dcSTX, and STX.

Clam Sample with GTXs and STX

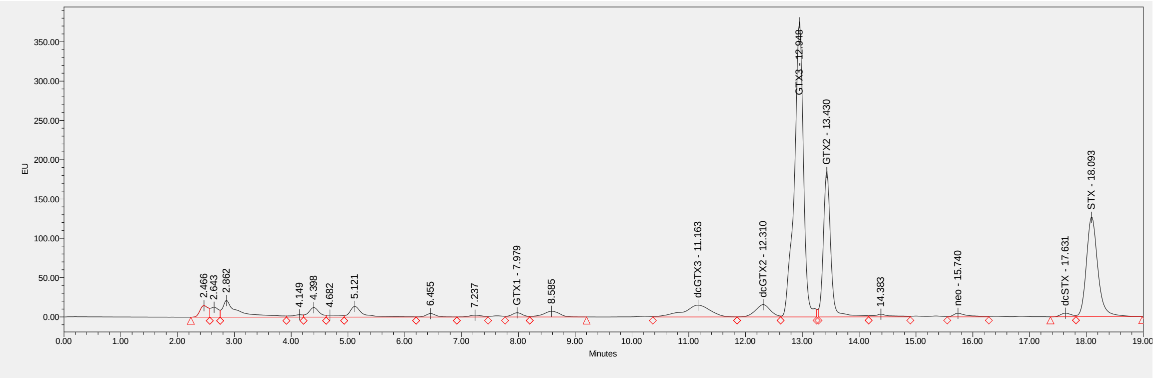

Figure S2: A HPLC chromatogram from a clam sample that contained quantifiable amounts of GTXs and saxitoxin (STX).

C Toxins Standards Mix

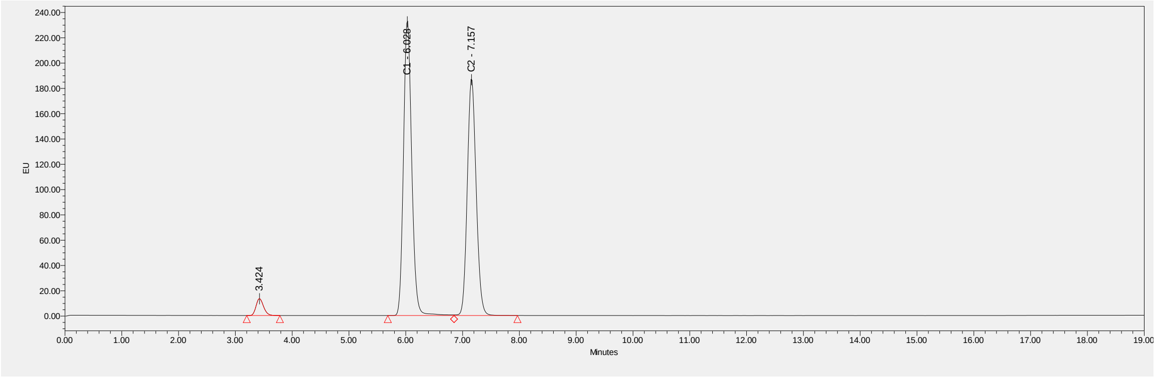

Figure S3: A HPLC chromatogram showing the peaks of the C toxin (C1 and C2) standards used in this study. Standards used were Certified Reference Material (CRM) obtained from National Research Council Canada (NRCC).
